# Behavioral lateralization in bipolar disorders: a systematic review

**DOI:** 10.1186/s40345-023-00320-9

**Published:** 2023-12-01

**Authors:** Annakarina Mundorf, Jette Borawski, Sebastian Ocklenburg

**Affiliations:** 1 ISM Institute for Systems Medicine and Department of Human Medicine, MSH Medical School Hamburg, Am Kaiserkai 1, 20457 Hamburg, Germany; 2 Division of Cognitive Neuroscience, Department of Neurology, Johns Hopkins University School of Medicine, Baltimore, MD USA; 3 Department of Psychology, Medical School Hamburg, Hamburg, Germany; 4 ICAN Institute for Cognitive and Affective Neuroscience, Medical School Hamburg, Hamburg, Germany; 5 Faculty of Psychology, Institute of Cognitive Neuroscience, Biopsychology, Ruhr University Bochum, Bochum, Germany

**Keywords:** Asymmetry, Handedness, Affective, Mania, Depression, Dichotic listening

## Abstract

**Background:**

Bipolar disorder (BD) is often seen as a bridge between schizophrenia and depression in terms of symptomatology and etiology. Interestingly, hemispheric asymmetries as well as behavioral lateralization are shifted towards a tendency of left-side or mixed-side bias in schizophrenia whereas no shift is observed in subjects with depression. Given the role of BD with both, (hypo)manic and depressive episodes, investigating hemispheric asymmetries in subjects with BD is an interesting objective.

**Method:**

A systematic review of studies including measures of behavioral lateralization in the form of handedness, footedness, eyedness, and language lateralization was performed resulting in 25 suitable studies.

**Results:**

A broad variety of methods was used to assess behavioral lateralization, especially for eyedness, footedness, and language lateralization hindering the integration of results. Additionally, for hand preference, studies frequently used different cut-off scores and classification systems. Overall, studies do not support alteration in side preference in BD subjects. Studies focusing on differences in handedness demonstrate that subjects show equal rates of right- and non-right-handedness as the general population. Few studies focusing on manic episodes point towards increased left-side bias in ear and eye dominance, but the small sample sizes and conflicting results warrant further investigation.

**Conclusion:**

The results reinforce that some disorders, such as BD, should not be treated as a homogenous group but sub-groups should be analyzed within the patient’s population. Particularly, clinical implications resulting from neuroimaging studies highlight the need to study hemispheric asymmetries given that they may be important to consider for brain stimulation protocols.

## Background

Bipolar disorder (BD) is sometimes described as a bridge between the diagnostic classes of schizophrenia and depression in terms of symptomatology and etiology (American Psychiatric Association 2013). This is especially interesting regarding neuronal alterations where research on BD may give important insights into our understanding of mania and depression and may thus indeed bridge the gap in current knowledge. Whereas depression and schizophrenia both have been extensively studied in neuroscience (a search on PubMed with the keyword ‘neuroscience AND (depression OR schizophrenia)’ revealed over 55.000 articles, https://pubmed.ncbi.nlm.nih.gov/?term=neuroscience+AND+%28depression+OR+schizophrenia%29, accessed on 17 Oct. 2023), less research is found on BD (a search for ‘neuroscience AND bipolar disorders’ on PubMed revealed around 7.600 articles, https://pubmed.ncbi.nlm.nih.gov/?term=neuroscience+AND+bipolar+disorders, accessed on 17 Oct. 2023). Especially in the context of the clinical neuroscience of lateralization, far less is known about BD than about either depression or schizophrenia (Mundorf and Ocklenburg 2021). Of note, the inherent functional, structural, and molecular asymmetries between the hemispheres pose a general organizational principle across species and enable our brain to be most efficient (Ocklenburg et al. 2022; Ocklenburg and Güntürkün 2017; Ocklenburg and Mundorf 2022; Vallortigara and Rogers 2020).

Recently, a group of researchers performed a review on cerebral asymmetries in BD subjects given the proposed role of cerebral asymmetry in the pathophysiology of BD (Moebus et al. 2023). To this end, they summarized resting-state and task-based functional cerebral asymmetries in manic and depressive episodes in BD subjects. Interestingly, the findings highlight a left-hemispheric dominance in the frontal lobe during manic episodes but a right-hemispheric dominance in regions of the frontal and temporal lobe in BD depression. The researchers further underline that these differential asymmetries in BD are important to consider for brain stimulation protocols and thus warrant further investigation (Moebus et al. 2023). While these findings are important, behavioral asymmetries were not included in this analysis, making it unclear whether behavioral asymmetries are altered in BD or not.

Atypical asymmetries in subjects diagnosed with schizophrenia have been widely studied indicating pronounced alteration compared to healthy controls (Gutman et al. 2022; Mundorf et al. 2021). For example, meta-analyses confirmed an overall decrease in the typically leftward asymmetry of the planum temporale in schizophrenia (Shapleske et al. 1999; Sommer et al. 2001). Interestingly, others demonstrated that the individual degree of decreased lateralization in the planum temporale showed a positive correlation with symptom severity (Oertel et al. 2010), reinforcing a direct link between atypical lateralization and schizophrenia symptoms. Of note, a recent automated voxel-based morphometry study found significant differences in gray matter asymmetry in subjects diagnosed with schizophrenia compared to BD subjects and also when contrasting the subjects to healthy controls (Pinto et al. 2023) hinting at differentially atypical asymmetries between the disorders.

Hemispheric asymmetries have also been largely studied in individuals diagnosed with depression or mood disorders (Gibson et al. 2022; Mundorf et al. 2021). Especially as the amygdala, a cluster of neural nuclei involved in emotion processing, is known to show a strong rightward structural hemispheric asymmetry (Kirstein et al. 2023; Ocklenburg et al. 2022). Importantly, studies from Finland and China report increased volume of the left amygdala in subjects diagnosed with depression whereas controls did not show a volume asymmetry (Mervaala et al. 2000; Xia et al. 2004).

These hemispheric asymmetries are reflected in behavioral asymmetries, also called behavioral lateralization (Ocklenburg and Güntürkün 2017). Generally, different forms of behavioral lateralization such as handedness, footedness, and eyedness show strong correlations with each other as well as correlations with measures of mental health in the general population (Mundorf et al. 2023). For example, individuals with a higher tendency toward a left-side bias demonstrated higher scores in several negative dimensions such as stress reactivity, burnout, and other mental health measures (Mundorf et al. 2023).

In the general population, around 80–90% of individuals are right-handers whereas the frequency of left-handedness lies between 9.3% (according to stringent criteria when assessing left-handedness) to 18.1% for the broader definition of non-right-handedness, i.e., individuals favoring the left hand or use both hands equally often (Papadatou-Pastou et al. 2020). In line with the hypothesis that atypical hemispheric asymmetries are associated with atypical behavioral lateralization (i.e., higher prevalence of non-right-side bias), higher rates of non-right-handedness are found in several mental disorders such as attention deficit hyperactivity disorder (Nastou et al. 2022), autism spectrum disorders (Markou et al. 2017), post-traumatic stress disorder (Borawski et al. 2022), and schizophrenia (Dragovic and Hammond 2005; Hirnstein and Hugdahl 2014). One large cohort study even found an increased prevalence of 42.4% in subjects diagnosed with schizophrenia for non-right-handedness and 34.1% for mixed-handedness (Mallet et al. 2022a). Furthermore, this study demonstrated that mixed-handedness was significantly associated with increased positive symptoms and a current depressive episode (Mallet et al. 2022a). However, in individuals diagnosed with depression or depressive symptoms, the rates of left-, mixed- or non-right-handedness did not differ from the general population according to a meta-analysis (Packheiser et al. 2021).

Another form of behavioral lateralization that has been extensively studied in schizophrenia is language lateralization assessed with the dichotic listening paradigm. In this paradigm, the participant is presented with two different stimuli simultaneously played to both ears over headphones (Hugdahl 2011; Westerhausen and Kompus 2018). Most paradigms use language stimuli like consonant-vowel syllables enabling the assessment of language lateralization (Hugdahl 2011; Westerhausen and Kompus 2018). A meta-analysis of existing studies revealed that subjects diagnosed with schizophrenia have weaker language lateralization than healthy controls. This was especially pronounced in subjects experiencing auditory hallucinations (Ocklenburg et al. 2013).

In terms of language lateralization, a review on negative affect and depression demonstrates that subjects with clinical depression show an atypically large right ear advantage in the verbal dichotic listening task that was even predictive of the individual response to pharmacological treatment (Gadea et al. 2011).

Consequently, these interesting results of atypical behavioral lateralization found in subjects diagnosed with schizophrenia or depression now pose the question of whether atypical lateralization is also present in subjects diagnosed with BD. Studies investigating ear dominance in BD subjects with the use of the dichotic listening paradigm report an increased left ear advantage during manic episodes but a ‘typical’ (as seen in controls) right ear advantage during recovery (Kaprinis et al. 1995) whereas others do not find significant differences between BD subjects and controls (Force et al. 2008; Green and Walker 1986). In terms of eye dominance, some studies suggest an increased left eye dominance in the hole test in BD subjects compared to controls (Goodarzi et al. 2015; Shan-Ming et al. 1985). In this test, participants are asked to look at an object through the hole in e.g., a card to establish the preferred eye (Gur 1977). Studies on foot preference seem to reveal mixed results with either increased lateralization (Savitz et al. 2007) or decreased left lateralization (Atagun et al. 2012) in BD subjects.

Regarding studies in handedness in subjects diagnosed with BD, one of the larger studies (*N* > 100) reported increased rates of non-right-handedness in BD compared to controls (van Dyck et al. 2012). But a much larger study with over 1000 BD subjects found no difference in hand preference compared to controls (Mallet et al. 2022b). However, as most of these studies include small sample sizes, different methods to assess the behavioral measure, and different types of BD (type I or type II), a systematic analysis and integration are essential before any final conclusions can be drawn. Given the interesting dimension of BD with manic and depressive episodes as well as differences in these episodes represented in a type I and type II BD diagnosis, differential results depending on the type of BD or the current episode experienced by the subjects, are to be expected.

But as of now, a systematic integration of findings on behavioral lateralization in BD is missing even though this understanding could provide useful insights into the neuroscience of the disorder. This systematic review aims to summarize the current knowledge of behavioral lateralization in BD subjects in terms of handedness, footedness, eyedness, language lateralization (assessed with the dichotic listening paradigm) and behavioral markers of visuospatial attention and visual perceptual asymmetries. When possible, we try to highlight the type of BD studied as well as the current episode of subjects (manic or depressive) to further disentangle potentially differential effects of BD. Finally, the review aims to provide insights into the important role of behavioral lateralization in mental disorders and to highlight the clinical implications derived from it.

## Methods

**Fig. 1:**
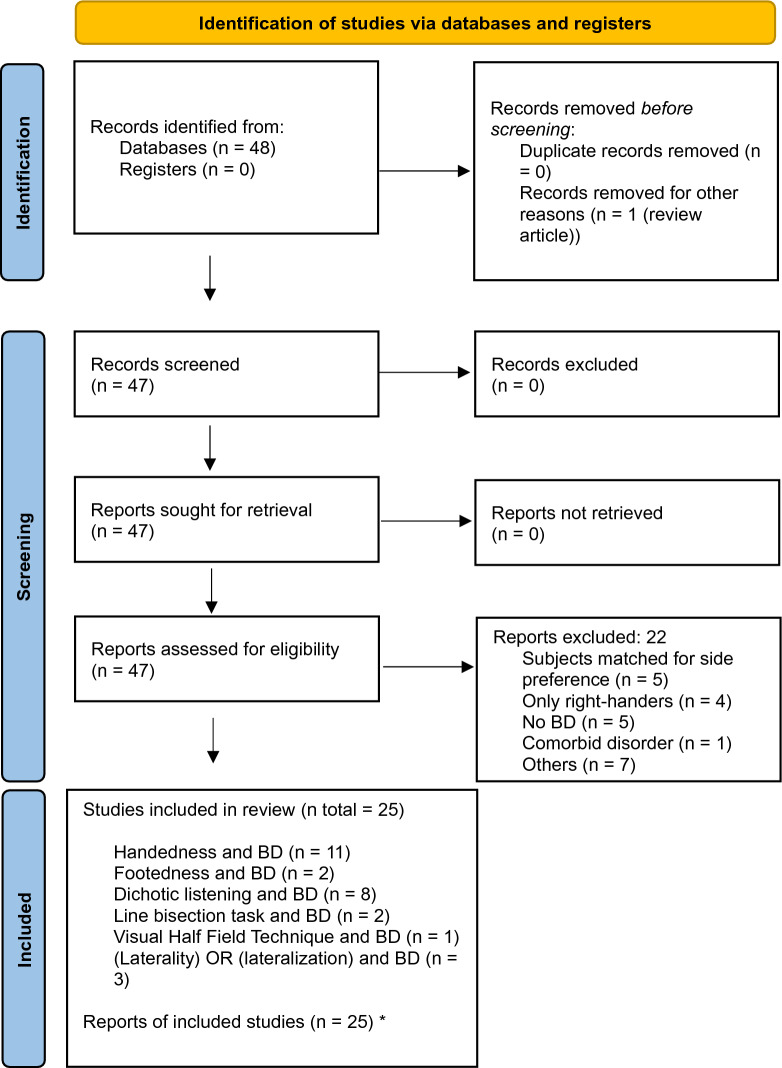
PRISMA flow diagram depicting the process of identifying, screening, and inclusion of the literature.  *Two studies appeared during two different searches (with different key words), as they included more than one suitable measure (Adapted from Page et al. (2021))

A literature search was conducted between January 2023 and May 2023. The databases PubMed (https://pubmed.ncbi.nlm.nih.gov/), PubPsych (https://www.pubpsych.eu/), ResearchGate (https://www.researchgate.net/), and Google Scholar (https://scholar.google.com/) were screened using the keywords: ((Handedness) OR (hand preference) OR (footedness) OR (foot use) OR (dichotic listening) OR (line bisection task) OR (visual half field technique) OR (laterality) OR (lateralization)) AND ((bipolar disorder) OR (manic depression)). Study identification was performed in multiple steps as different keyword combinations were searched separately. No automation tool was used. First, titles and abstracts were screened, and reviews and meta-analysis (n = 1) were removed, resulting in a total of n = 47 potentially relevant studies. Inclusion criteria were (i) a diagnosis of BD, (ii) articles must contain information on either handedness, footedness, dichotic listening, line bisection task or the visual half field technique, (iii) data must be given for BD subjects separately, (iv) original research article in English language. Exclusion criteria were (i) review articles, (ii) BD subjects and controls were matched for side preference, (iii) article only included right-handers, (iv) no BD subjects, (v) comorbid disorder. After the full-text screening, another n = 22 studies were removed due to inclusion and exclusion criteria leading to 25 articles included in this review. This process is presented in the flow chart following Prisma Guidelines (Page et al. 2021) in Fig. 1.

## Results

A total of 25 studies are included in this review. Some studies included information on more than one form of asymmetry and thus eight studies report results on dichotic listening, seven on eyedness, 17 on handedness, and three on footedness in BD subjects. For details see Table 1.

**Table 1:** Summary of studies including measures of behavioral lateralization in subjects with bipolar disorder (BD) and healthy controls (HC)

Lateralized behavior	Study	Sample	Type of BD	Method laterality	Results for BD
Dichotic listening	Bruder et al. (1981)	BD: 14; HC: 15, all RH	BD I + II	DL click detection	↓ Threshold intensity when right ear click preceded left ear click
	Green and Walker (1986)	BD: 16; HC: 12	BD I	DL digits	↔
	Bruder et al. (1989)	BD:22, HC: 30	BD II	DL consonant-vowel + tone	↔
	Bruder et al. (1992)	BD: 11, HC: 24	BD	DL consonant-vowel + tone	↔
	Kaprinis et al. (1995)	BD: 26, HC: 30 All RH	BD	DL digits	LEA during manic episode; REA in recovery
	Force et al. (2008)	BD: 18; HC: 36	BD	DL tones	↔
	Bozikas et al. (2014)	BD: 20; HC: 35	BD I	DL two-syllable words	↓ REA
	Najt and Hausmann (2014)	BD: 22; HC: 18	BD I	DL emotional prosody + linguistic	↔ Linguistic DL ↓ Advantage in emotional prosody DL
Eyedness	Shan-Ming et al. (1985)	BD: 56; HC: 432	Manic-depressive	Hole test	↑ Left-eye dominance
	Atagun et al. (2012)	BD: 68; HC: 65	BD I	Near-far alignment and kaleidoscope tests	↔
	Goodarzi et al. (2015)	BD: 17; HC: 113 (high-school)	BD	Hole test	↑ Left-eye dominance
Asymmetries in visual modality	Bruder et al. (1989)	BD: 22; HC: 30	BD II	Visual half field, dot enumeration	↓ Performance for left visual field
	Bruder et al. (1992)	BD: 11; HC: 24	BD	Visual half field, dot enumeration	↓ Performance for left visual field
	Rao et al. (2010)	BD: 31; HC: 103	Bipolar affective, in remission	Line bisection	Leftward deviation with RH
	Najt et al. (2013)	BD: 22; HC: 18	BD I	Line bisection	No leftward bias with LH versus RH
Handedness	Shan-Ming et al. (1985)	BD: 56; HC: 432	Manic-depressive	Tool use	↔
	Bruder et al. (1989)	BD:22; HC: 30	BD II	EHI LQ (2-categorial)	↔
	Bruder et al. (1992)	BD: 11; HC: 24	BD	EHI LQ (2-categorial)	↔
	Clementz et al. (1994)	BD: 36; HC: 33	BD	EHI (2-categorial)	↔
	Koek et al. (1999)	BD: 13	BD I + II	EHI	↑ Ratio of LH
	Noga et al. (2001)	BD: 12; HC: 34 Twins	BD	EHI (2-categorial)	↔
	Kravariti et al. (2005)	BD: 30; HC: 30	BD I	AHQ	↑ Ratio of LH
	Sanches et al. (2005)	BD: 15; HC: 21 (children-adolescents)	BD euthymic	Not specified	↔
	Savitz et al. (2007)	BD: 78; HC: 66	BD I + II	WHQ	↑ Lateralization, ↔ side
	Fasmer et al. (2008)	BD I: 36; BD II: 51	BD I + II	EHI (3-categorial)	↔
	Nowakowska et al. (2008)	BD: 796	BD I + II	EHI (2-categorial)	↔
	Atagun et al. (2012)	BD: 68; HC: 65	BD I	EHI, peg board	↑ RH, ↓ reaction time
	van Dyck et al. (2012)	BD: 155; HC: 179	BD	EHI + self-report	↑ Non-right-handedness
	Goodarzi et al. (2015)	BD: 17; HC: 113 (high-school)	BD	EHI (3-categorial)	↔
	Van Voorhis et al. (2019)	BD: 21; HC: 56	BD I	EHI (sum score) + writing hand	↔
	Cansiz and Ince (2021)	BD: 66; HC: 66	BD I euthymic	EHI LQ	↔
	Mallet et al. (2022b)	BD I: 1162; BD II: 1012	BD I + II	Self-report	↔
Footedness	Savitz et al. (2007)	BD: 78; HC: 66	BD I + II	WFQ	↑ Lateralization, ↔ side
	Atagun et al. (2012)	BD: 68; HC: 65	BD I	Not specified	↓ Left-lateralized
	Goodarzi et al. (2015)	BD: 17; HC: 113 (high-school)	BD	Chapman foot preference inventory	↔

In case the type of BD is not further specified, the table only reads ‘BD’

*LH* left hand, *RH* right hand, *AHQ* Annett Handedness Questionnaire, *DL* dichotic listening, *EHI* Edinburgh Handedness Inventory, *LEA* left-ear advantage, *REA* right-ear advantage, *WHQ* Waterloo Handedness Questionnaire, *WFQ* Waterloo Footedness Questionnaire

↑: increased/stronger; ↔: no difference; ↓: decreased/weaker compared to controls

### Hemispheric asymmetries for acoustic stimuli in bipolar disorder

Hemispheric asymmetries for acoustic stimuli can be assessed with the dichotic listening paradigm in which the participant is presented with two different stimuli simultaneously played to both ears over headphones. The participant then has to indicate which of the two stimuli they heard. Therewith, researchers can establish whether the subject shows advantage of one ear for processing stimuli (Westerhausen and Kompus 2018). Overall, there are different versions of this paradigm, with most of them using language stimuli like consonant-vowel syllables and thus assessing language lateralization. However, there are also variants with non-language stimuli. For example, the threshold intensity needed for dichotic click detection was measured in 14 right-handed BD subjects (BD I + II) and 15 right-handed healthy controls (Bruder et al. 1981). Here, BD subjects showed greater signs of reversed lateral asymmetry, meaning that the threshold intensity was lower when the right ear click preceded the left ear click. Consequently, the lateral asymmetry (RL minus LR scores) significantly differed from the controls. When correlating scores of depression and laterality, greater severity of depressive or endogenous symptoms was associated with less lateral asymmetry in BD subjects (Bruder et al. 1981). In contrast, Green and Walker did not find differences in dichotic listening performance when two different inputs (strings of three digits) were presented simultaneously when analyzing 16 BD subjects with mania compared to controls (Green and Walker 1986). Others tested 22 BD type II subjects (one with BD type I) in a dichotic listening paradigm with consonant-vowel discrimination and a complex tone test (Bruder et al. 1989). Here, no differences between BD subjects and healthy controls were found. In a follow-up study, 11 BD subjects were again tested with the dichotic consonant vowel test, followed by the complex tone test (Bruder et al. 1992). Again, no difference between groups was evident. In a verbal dichotic listening test for pairs of digits, 26 BD right-handed subjects were tested first during an acute manic episode and then, after recovery (Kaprinis et al. 1995). During the manic episode, BD subjects demonstrated a left-ear advantage in contrast to the found right-ear advantage in healthy controls. Upon recovery, the left-ear advantage shifted towards a right-ear advantage (Kaprinis et al. 1995). In another study, 18 BD subjects were assessed for language lateralization with a space and pitch dichotic listening task and had to identify specific tones (pips). Here, BD subjects did not differ in side preference from healthy controls (Force et al. 2008). Bozikas et al. (2014) applied dichotic listening using two-syllable words as verbal stimuli to 20 BD type I subjects compared to controls at the beginning of hospitalization and on the day before discharge. Here, a non-forced versus forced condition was included where participants are asked to repeat all the words they hear (non-forced) or asked to repeat only the left or the right ear stimuli (forced left/right) while in both conditions different stimuli are played simultaneously to both ears (Hugdahl 2011; Westerhausen and Kompus 2018). BD subjects with affective psychosis did not demonstrate a right-ear advantage in the non-forced condition. In the forced conditions, subjects were able to focus on the corresponding ear but with less success than controls. The results did not change between time points (Bozikas et al. 2014). Differences in ear advantage were furthermore analyzed in 22 BD type I subjects in an emotional prosody task and a linguistic dichotic listening task and compared to healthy controls (Najt and Hausmann 2014). BD subjects demonstrated no ear advantage for emotional prosody whereas controls showed a left-ear advantage. In the linguistic condition, both groups revealed a right-ear advantage.

So far, results on altered hemispheric asymmetries for acoustic language stimuli and other acoustic stimuli in BD subjects are mixed with several studies reporting no difference from controls and some reporting an increased left-ear advantage, especially in manic episodes. However, there is great heterogeneity in tasks performed with the dichotic listening paradigm that exacerbates the interpretation of results.

### Eyedness and behavioral markers of visuospatial attention and visual perceptual asymmetries in bipolar disorder

There are several established tests to examine potential differences in eye preference (Ocklenburg and Güntürkün 2017). Some studies use a so-called ‘hole test’ where the participants are asked to look at an object through the hole of e.g., a card to establish the preferred eye (Gur 1977). In this assessment method, the subject is asked to look through a hole in e.g., a card with both eyes at a distant object. Then, they are asked to move the card closer to the face while the experimenter observes which eye shows a dominance for looking through the card (to which eye the subject moves the card or which eye is closed). In line with this, one of the oldest studies examined 56 manic-depressive subjects with psychosis in the hole test to assess eye dominance and revealed increased rates of left eye dominance in subjects compared to controls (Shan-Ming et al. 1985). Goodarzi et al. (2015) analyzed eye dominance with a “looking through a hole” method in 17 BD subjects and found a higher prevalence of left-eye dominance compared to healthy controls as well.

Eye dominance was also analyzed in 68 BD type I subjects and 65 healthy controls using the near-far alignment and kaleidoscope tests. In this test, a near point is defined as the tip of a stick placed 40 cm away from the participants face while they jaw is placed on a supporting apparatus. The second, far point is marked on a wall or screen 3 m further away than the first point. The subject is asked to focus both eyes on the far point with the first point set as reference point. Then, the participant is asked to close one eye first, then change the eye closed. If the close point shifts from the far point in the horizontal line when one of the eyes is closed, the closed eye is set as the dominant eye. With this test, the research team found no difference between BD subjects and controls in eye dominance (Atagun et al. 2012).

Taken together these studies point towards a increased left-eye dominance in BD compared to healthy controls. It is to note that the different methods used lead to contradicting results, e.g., the two studies reporting stronger left-eye dominance used a different method to assess eyedness (Goodarzi et al. 2015; Shan-Ming et al. 1985) compared to the other study.

In addition to tests that assess eyedness, other behavioral markers of asymmetries in the visual modality have been assessed in BD subjects.

In the above-mentioned (3.1.) study by Bruder et al. (1989), the 22 BD type II subjects (one with BD I) were not only assessed in terms of dichotic listening but also in the dot enumeration task to investigate visual perceptual asymmetries. The BD subjects demonstrated no left-visual field advantage and generally poorer performance for the left visual field compared to controls (Bruder et al. 1989). In a follow-up study, 11 BD subjects were again examined in the visual half-field paradigm (Bruder et al. 1992). Again, the BD subjects did not show the expected left visual field (right hemisphere) advantage for dot enumeration that was evident in healthy controls. In addition, BD subjects demonstrated poorer performance for the left visual field but not for the right visual field stimuli (Bruder et al. 1992).

A study applying the line bisection task, a common measure of asymmetries in visuospatial attention (Barnett 2006), in 31 BD subjects found that these demonstrated a leftward deviation when using their right hand but healthy controls did not (Rao et al. 2010). Another study tested 22 BD subjects (euthymic at study start) in the line bisection task. Here, the BD subjects did not show a larger leftward bias with the left hand than with the right hand which was prominent in healthy controls as BD subjects did not significantly deviate from the veridical center of the lines (Najt et al. 2013).

In sum, studies assessing behavioral markers of visuospatial attention and visual perceptual asymmetries point towards a poorer performance for the left visual field in BD subjects compared to controls.

### Handedness

Several studies have focused on the objective to analyze potential differences in handedness in BD subjects. There are several established methods to assess hand preference, such as the Edinburgh Handedness Inventory (EHI) or the Annett Handedness Inventory (Annett 1970; Oldfield 1971). Both the EHI and Annett Handedness Inventory use several questions on tool use to assess the preferred hand. Based on the answers, a lateralization quotient (LQ) can be computed. According to Oldfield (1971), the LQ can be calculated with the formula LQ = ((R − L) / (R + L) × 100, with R meaning the number of right-hand preferences and L the number of left-hand preferences. Usually, the LQ ranges from 100 (completely right-handed) to -100 (completely left-handed) with individuals having a negative LQ being classified as left-handers. Then, cut off scores are set to define handedness categories depending on whether a two-, or three-categorical classification system is used: for a dichotomous classification system, individuals with a LQ from − 100 to 0 are set as left-handed and subjects with a LQ from 0 to 100 as right-handed. Several different three-category systems have been proposed. For example individuals with a LQ ranging from − 100 to − 40 can be classified as left-handed, subjects with a LQ ranging from − 40 to 40 as mixed-handed and people with a LQ between 40 and 100 as right-handed (Thomas et al. 2023). However, other versions such as a cut-off of LQ = − 60 to 60 for mixed-handedness also have been used. Generally, both assessing and scoring of the EHI can be done following a variety of different methods, which may result in problems when replicating existing results (Edlin et al. 2015; Yeung and Wong 2022).

One of the older studies investigated hand preference in 56 manic-depressive subjects and controls by asking the participants to mimic the use of ten different tools and tasks (Shan-Ming et al. 1985). Then, a dichotomous classification system was used by categorizing them into right-handers (all items performed only by the right hand) or left-handers (at least one item carried out by the left hand). There was no difference in handedness between manic-depressive subjects and controls but a trend toward increased rates of left-handedness in male BD subjects compared to male controls (Shan-Ming et al. 1985). Others have investigated 22 BD type II subjects (one with BD I) for differences in LQ with the help of the EHI (Bruder et al. 1989). The researchers found no overall difference in LQ between groups (bipolar melancholic subjects: mean LQ of 63.9, SD: 56.7; atypical bipolar subjects: Mean LQ: 44.0, SD: 74.6; controls: Mean LQ: 74.5, SD: 42.0). In a follow-up study, 11 BD subjects with a history of hypomania were again tested with the EHI, and LQs were calculated. Again, the groups did not differ in hand preference (BD subjects: Mean LQ 53.5, SD: 63.4; controls: Mean LQ: 71.5, SD: 42.4) (Bruder et al. 1992). One study analyzed handedness with the EHI and resulting sum scores with 10 equaling most left-handed to 50 meaning most right-handed but found no difference in handedness between the 36 BD subjects with a first-lifetime experience of a psychotic episode and 33 healthy relatives (Clementz et al. 1994). In a further study, handedness was also investigated using the EHI in 13 BD subjects (11 with BD type I) resulting in ten subjects classified as right-handers and three as left-handers. Interestingly, all left-handers had BD type I (Koek et al. 1999). Noga and colleagues examined handedness in BD in a monozygotic twin design with the EHI. The researchers used the EHI to define handedness and found no difference in handedness between groups with four twin BD pairs being concordant right-handers and two BD pairs mixed-handers. In health twins, 15 pairs were right- and two mixed-handed (Noga et al. 2001).

Handedness was further investigated with the Annett Handedness Questionnaire and a two categorial classification system in 30 healthy controls and 30 BD type I subjects, of whom 15 had predominant current clinical depression and 15 predominant current clinical mania. In the BD type I predominantly mania group, 12 subjects were right-handed and three left. In the predominantly depression group, 11 subjects were right- and four left- handed. In the control group, 25 individuals were right-handed and five left-handed. When comparing the results, it seems that the frequency of left-handedness was increased in BD subjects compared to controls (Kravariti et al. 2005).

In a study with 15 children and adolescents diagnosed with BD and 21 healthy controls, no difference in handedness was found with 2 BD subjects and 1 control being left-handed (Sanches et al. 2005). Of note, 14 BD subjects were euthymic, and one was mildly depressed at the time of the investigation. Unfortunately, the study did not specify how handedness was assessed (Sanches et al. 2005). In a sample of 55 BD type I subjects, 23 BD type II subjects and 66 unaffected relatives hand preference was assessed with the Waterloo Handedness Questionnaire (WHQ). Here, scores obtained from the WHQ ranged from − 72 to + 72 with the absolute values indicating the strength of lateralization. BD subjects were significantly more lateralized compared to healthy relatives but there was no difference in hand preference (BD I: 13% left-handed; BD II: 9% left-handed; controls: 9% left-handed) between groups (Savitz et al. 2007). Fasmer and colleagues (2008) examined hand preference with a three-categorical system by applying the EHI and found that, in 36 BD I subjects, 78% were right-handed, 6% left- and 17% mixed-handed whereas in a total of 51 BD type II subjects, 59% were right-, 8% left- and 33% mixed-handed. Overall, there was no significant difference in rates of non-right-handedness between these groups nor compared to participants with depression (Fasmer et al. 2008). But compared to the general population, the rates of mixed-handedness are increased in both BD types (mixed-handers estimate in the general population: 9.33%, Papadatou-Pastou et al. (2020)). Differences in LQ assessed with the EHI were analyzed in 796 BD type I or II subjects (74.4% BD I). Here a two-categorical classification system was used with positive LQ scores indicating right-handedness and negative scores for non-right-handedness. Overall, 15% of BD subjects were non-right-handed (Nowakowska et al. 2008). Of note, the prevalence of non-right-handedness in the general population is estimated at 18.10% (Papadatou-Pastou et al. 2020). Thus, the prevalence of non-right-handedness was not significantly increased in the BD subjects when compared to recent meta-analytical estimates.

Hand preference and performance were assessed in 68 BD type I subjects and 65 healthy controls with the EHI and the nine-hole peg board test, respectively (Atagun et al. 2012). The study demonstrates that euthymic BD subjects show significantly higher scores in the EHI (M: 84.41, SD: 12.02) compared to controls (M: 79.46, SD: 13.55) and needed more time to complete the peg boars test for both hands. Moreover, the study revealed a significant positive correlation between lateralization and processing speed in BD subjects that was not found in healthy controls (Atagun et al. 2012). Non-right-handedness was also assessed with the 10-item EHI and additionally by subjects’ self-identification of handedness in 155 adolescent and adult BD subjects (48% euthymic, 26% depressed, and 26% elevated mood) and 179 healthy controls (van Dyck et al. 2012). Overall, BD subjects had lower EHI scores and were significantly more non-right-handed (15.4%) compared to the controls (7.3%). This difference was especially pronounced in adolescent BD subjects (20.0%) when compared to healthy adolescents (5.7%). Interestingly, mood state had no significant effect on handedness. Others analyzed handedness with the EHI and a three-categorical system (left-handed: 10 to − 5; ambidextrous: − 4 to + 7; right-handed: + 8 to + 10) in 17 high-school BD subjects and found no overall difference in hand preference compared to 113 healthy controls (Goodarzi et al. 2015). In a study including BD type I subjects, the sum scores of the EHI as well as the preferred hand for writing was assessed in 21 BD type I subjects and 56 healthy controls. Comparing the results revealed no statistical difference in sum scores (BD: 44.2; controls: 40.7) nor in the ratio of hand preference for writing (BD: 0 left-, 1 mixed-, 20 right-handers; Controls: 8 left-, 0 mixed- and 48 right-handers) (Van Voorhis et al. 2019). Hand preference was furthermore investigated in 66 BD type I subjects in a current euthymic episode and 66 healthy controls. To this end, the EHI was applied and the LQ calculated ranging between + 100 and − 100 with negative scores indicating a greater predisposition to left-handedness, and a positive score of a right-handed preference. Again, no statistically significant difference in the prevalence of hand preference was found between BD (Mean LQ: 76.82, SD: 40.02) and control (Mean LQ: 64.32, SD: 48.22) subjects (Cansiz and Ince 2021). In a large-scale study including 1162 subjects with BD type I and 1012 subjects with BD type II, differences in handedness were measured by self-report with the single question if the participant is left, mixed, or right-handed. The comparison revealed no difference in rates of mixed- (2.4%) or non-right-handedness (11.6%; mixed- and left-handers together) between types of BD nor when compared to rates found in the general population. Interestingly, non-right-handedness was associated with a younger age of BD onset (Mallet et al. 2022b).

Overall, studies focusing on differences in handedness or hand preference in BD subjects demonstrate that BD subjects show the same rates of right- and non-right-handedness as the general population. Only one study even found higher scores in the EHI indicating more right-handedness in euthymic subjects (Atagun et al. 2012) whereas this study also revealed lower scores in the EHI and higher rates of non-right-handedness in BD subjects when compared to internal controls (Atagun et al. 2012). Contrarily, the study by van Dyck et al. (2012) highlights significantly increased rates of non-right-handedness especially in adolescent BD subjects (20.0%). Kravariti et al. (2005) further report increased frequency of left-handedness in BD subjects. However, in the broader picture is seems like there might be no difference in rates of hand preference in BD subjects. But, given that studies used different assessment methods and classification systems (some studies only differentiated between right-handers vs. non-right-handers while others differentiated between three categories: left-, mixed- or right-handers), comparing results across studies is hindered.

### Footedness

So far, only three studies have investigated footedness in BD. To this end, questionnaires are used, which ask the participant about which foot they mostly use for different tasks such as kicking a ball, picking up a marble, smoothing sand or stomp an insect. Then, the percentage of choosing the right or left foot is calculated (Chapman et al. 1987).

When analyzing foot preference with the Waterloo Footedness Questionnaire in the above-mentioned sample of 55 BD type I and 23 type II subjects, again, subjects with BD were significantly more lateralized compared to healthy relatives but overall did not show a significantly different favor of one side (left or right) (Savitz et al. 2007). Footedness was also assessed in 68 BD type I subjects and 65 healthy controls showing that BD subjects were significantly less left-lateralized than controls (Atagun et al. 2012). Unfortunately, the assessment of footedness is not further specified in the study. The above-mentioned study by Goodarzi et al. (2015) also investigated footedness with the Chapman Foot Preference Inventory in 17 BD subjects and found no difference in foot preference compared to healthy controls.

## Discussion

The study aimed to unravel potential differences in behavioral lateralization in BD subjects compared to the healthy population. To this end, studies investigating handedness, footedness, eyedness, and language lateralization in subjects diagnosed with BD were integrated and compared. So far, four main results can be conceived:

First, across all forms of behavioral lateralization, no overall difference in terms of side preference was prevalent with most studies reporting similar side tendencies as healthy controls (see Table 1). Interestingly, there seems to be an increased left ear advantage (Kaprinis et al. 1995) as well as a left eye advantage (Goodarzi et al. 2015; Shan-Ming et al. 1985) especially in manic episodes but the small sample sizes and conflicting results warrant further investigation.

Second, the broad variety of methods used to assess behavioral lateralization, especially for eyedness, footedness, and language lateralization makes the integration of results difficult. Additionally, for hand preference, studies frequently used different cut-off scores and classification systems (either two- or three-categorical) hindering the synthesis of results. In terms of handedness, it is especially important to differentiate between mixed-handers (marked by doing some tasks with the left and some with the right hand) and ambidextrous (e.g., writing with both hands equally) individuals which may not always have been the case in the included studies.

Third, studies raise the question of to what proportion handedness may be linked to neurodevelopmental aspects given that some studies included adolescent subjects with BD or different ages of BD onset (van Dyck et al. 2012; Goodarzi et al. 2015; Mallet et al. 2022b). For example, Goodarzi et al. (2015) found increased left-eye dominance in adolescent subjects with BD, but no difference in hand or foot preference. Contrarily, van Dyck et al. (2012) reported increased rates of non-right-handedness that were especially pronounced in adolescent BD subjects. Higher rates of non-right-handedness were linked to early onset BD in the study by Mallet et al. (2022b) reinforcing neurodevelopmental aspects. Similar to depression, BD is a heterogenous disorder with one subgroup marked by neurodevelopmental aspects with earlier age at onset (Cross-Disorder Group of the Psychiatric Genomics Consortium 2019). Indeed, studies on handedness in neurodevelopmental disorders report higher rates of non-right-handedness in attention deficit hyperactivity disorder (Nastou et al. 2022), autism spectrum disorders (Markou et al. 2017), stuttering (Papadatou-Pastou et al. 2023) and dyslexia (Packheiser et al. 2023), reinforcing the role of neurodevelopmental aspects in non-right-handedness. Given the hypothesis of a chronological component, age of onset may be a relevant factor for detecting atypical asymmetries. The heterogeneity in terms of age of BD onset may thus be partly responsible for the mixed results across studies.

Fourth, unfortunately, most studies did not differentiate between the type of BD (type I or II), nor did they assess acute manic or depressive episodes when testing the subjects. Grouping the results from individuals with a current manic episode or BD type I diagnosis with individuals with a current depressive episode or a type II BD diagnosis potentially dilutes effects that would be more pronounced in manic episodes as seen in the neuroimaging data (Moebus et al. 2023). Another possibility would be that individuals may show stronger left lateralization during manic episodes compared to depressive episodes and not a general atypical side preference. To this end, it would be important to analyze the degree of individual lateralization, for example by calculating the LQ, as this may give more meaningful results. But only a few included studies used the LQ to assess differences in hand preference (Bruder et al. 1989, 1992; Cansiz and Ince 2021). The meta-analyses of studies in individuals diagnosed with schizophrenia underline that there are differences in terms of language lateralization between acoustically hallucinating subjects and subjects without acoustic hallucinations (Ocklenburg et al. 2013). This reinforces that some disorders, such as BD and schizophrenia, should not be treated as a homogenous group regarding neuronal alterations, but that studies need to separate sub-groups within the patient’s population such as BD type I and type II or even more precisely, based on the current mood episode of the subjects.

The fact that overall, studies do not find a difference in handedness in BD type II or subjects currently in an euthymic mood does not come as a surprise given that no difference in handedness was found in depression either (Packheiser et al. 2021). However, it is important to note that animal models of affective disorders consistently report an association between increased leftward behavior and increased anhedonia and despair (Ecevitoglu et al. 2020; Farhang et al. 2014; Mundorf and Ocklenburg 2023; Soyman et al. 2018). Thus, it stands to reason that different symptoms of depression such as despair and anhedonia may be associated with atypical hemispheric asymmetries, but other symptoms of the diagnosis ‘depression’ are not. Again, sub-groups or symptom-based groups should be analyzed separately in depression to truly unravel the role of atypical lateralization in affective disorders.

In contrast, given the literature on schizophrenia and depression, one would have expected differences in ear dominance in subjects with BD as well. Considering that subjects diagnosed with schizophrenia have weaker language lateralization than healthy controls (Ocklenburg et al. 2013) and individuals suffering from clinical depression show an atypically large right ear advantage in the verbal dichotic listening task (Gadea et al. 2011). So why is there no overall shift in ear dominance in BD subjects similar to schizophrenia or depression? One simple explanation may be that only half of the studies assessing hemispheric asymmetries for acoustic stimuli in BD used a verbal dichotic listening paradigm that was also used in depression and schizophrenia possibly leading to contradicting results (Bozikas et al. 2014; Bruder et al. 1989, 1992; Najt and Hausmann 2014).

Unfortunately, most studies do not focus on manic episodes or did only include a few subjects diagnosed with BD type I, hindering the differentiation between manic and depressive mood states. One study focusing on both episodes consecutively found that subjects demonstrated a left-ear advantage during a manic episode and a right-ear advantage in recovery from the manic episode (Kaprinis et al. 1995). The study by Kravariti et al. (2005) also included solely subjects with BD type I and demonstrates an increased ratio of left-handedness in BD. Considering the strong left lateralization present in subjects diagnosed with schizophrenia, the above-mentioned studies, and the close relationship between the two disorders, further investigation of altered asymmetries in manic episodes will be especially interesting to the clinical neuroscience community. To better define subtypes of BD, we recommend including assessments of depressive and manic symptoms allowing for the analysis of associations on a symptom-based approach. This is likely to result in more clear data than only using categories based on diagnostic manuals (BD type I or II). Since this approach allows for including the current mood but also to analyze associations of specific symptoms and hemispheric asymmetries (such as language lateralization and psychotic symptoms in schizophrenia, Ocklenburg et al. 2013).

## Limitations

This review highlights some crucial limitations of the current lateralization research in BD. First, a broad variety of methods was used to assess behavioral lateralization in eyedness, footedness, and language lateralization hindering the integration of results. Secondly, especially for hand preference, studies frequently used different cut-off scores and classification systems (two- vs. three-categorial) making it difficult to compare findings. Third, most studies had quite small sample sizes which reduces the validity of results. Fourth, not all studies analyzed the data separately for the type of BD or the current mood state. But given the results from the studies that did differentiate between BD type or current mood state, this should be set as an additional experimental variable. Consequently, conflicting results derive that warrant further investigation.

Besides these aspects of the content and methods of the studies integrated in this systematic review, the used search strategy may be a further limitation, as other forms of behavioral asymmetries not explicitly mentioned in the search terms may have been overlooked.

## Conclusion

In conclusion, studies investigating handedness, footedness, eyedness, and language lateralization in subjects diagnosed with BD do not show overall alterations in behavioral preferences in BD. Few studies focusing on manic episodes point towards increased left ear and eye dominance, but the small sample sizes and conflicting results warrant further investigation. The results reinforce that some disorders, such as BD and schizophrenia, should not be treated as a homogenous group regarding neuronal alterations, but that studies need to separate sub-groups within the patient’s population such as BD type I and type II or even more precisely, based on the current mood episode of the subjects. Particularly, regarding clinical implications, results from neuroimaging studies reinforce the need to study hemispheric asymmetries given that they may be important to consider for brain stimulation protocols.

## Data Availability

Data sharing is not applicable to this article as no datasets were generated or analyzed during the current study.

## Abbreviations

BD: Bipolar disorders
EHI: Edinburgh Handedness Inventory
LQ: Lateralization quotient
WHQ: Waterloo Handedness Questionnaire
